# HTQC: a fast quality control toolkit for Illumina sequencing data

**DOI:** 10.1186/1471-2105-14-33

**Published:** 2013-01-31

**Authors:** Xi Yang, Di Liu, Fei Liu, Jun Wu, Jing Zou, Xue Xiao, Fangqing Zhao, Baoli Zhu

**Affiliations:** 1 CAS Key Laboratory of Pathogenic Microbiology and Immunology, Institute of Microbiology, Chinese Academy of Sciences, NO.1 West Beichen Road, Chaoyang District, Beijing, China; 2 Beijing Institutes of Life Sciences, Chinese Academy of Sciences, NO.1 West Beichen Road, Chaoyang District, Beijing, China

## Abstract

**Background:**

Illumina sequencing platform is widely used in genome research. Sequence reads quality assessment and control are needed for downstream analysis. However, software that provides efficient quality assessment and versatile filtration methods is still lacking.

**Results:**

We have developed a toolkit named HTQC – abbreviation of High-Throughput Quality Control – for sequence reads quality control, which consists of six programs for reads quality assessment, reads filtration and generation of graphic reports.

**Conclusions:**

The HTQC toolkit can generate reads quality assessment faster than existing tools, providing guidance for reads filtration utilities that allow users to choose different strategies to remove low quality reads.

## Background

Next generation sequencing technologies are generating massive sequence data [1], and different platforms can introduce varied level of sequence reads error. Among them, the Illumina platform is the most widely used for genome sequencing with the least error rate per base. However, due to the nature of the method, it still presents a considerable amount of errors that has its specific errors pattern [2]. The device performs sequencing by DNA synthesis on clusters of identical DNA molecules simultaneously. When elongation of some DNA molecules is stopped accidentally, it creates disturbance of the cluster’s fluorescent signal, resulting sequencing errors. Such errors accumulate during the process of sequencing, and cause reads quality decreasing while the length grows. Besides, deficiency on sequencing chips and the existence of air bubbles on chip surface can cause failure on reads from a whole tile. To get reliable result in downstream analysis, it is necessary to remove low quality reads avoiding mismatches in read mapping, and false paths during genome assembly.

Currently there are several reads quality control (QC) software tools (Table 1). However, all these software tools lack of function versatility or run-time efficiency. For example, PIQA and FastQC only create reports on reads quality, but provide no tool for reads filtration [3,4]. SolexaQA and BIGpre provide only read trimming utility [5,6]. Furthermore, many of these software tools are implemented using Perl language [3,5,6], which as a dynamic-typed script language, provides ease on developing mini programs, but causes severe loss on run-time performance [7]. Therefore, a high performance sequence reads QC toolkit is needed for a faster assessment and versatile functionality.

**Table 1:** Comparison of sequencing quality control software tools

Name	Programming language	Sequencing platforms	Reads filtration methods
HTQC	C++	Illumina	Tail trimming, filter by quality/length/tile
FastQC	Java	Not limited, any FASTQ file	No
NGS QC	Perl	Illumina, 454	Trimming only
SolexaQA	Perl	Illumina	Trimming only
BIGpre	Perl	Illumina, 454	Duplicate removal
PIQA	R, C++	Illumina	No

### Implementation

The HTQC toolkit consists of six programs that can perform reads quality assessment and filtration. To improve run-time performance, the time-consuming programs are implemented using C++. The FASTQ format is used for sequence data input and output, and the QC report is generated as tab-separated plain-text file [8]. To create graphical charts of QC report, a Perl script is included. The GNU Glib is used for base utilities such as command-line parser, portable support for threads and asynchronous queues. All programs of HTQC toolkit are capable of single-end or paired-end sequencing experiments, 33-based or 64-based quality score encodings and FASTQ sequence identifier format from different version of CASAVA tools (the traditional format like “@HWUSI-EAS100R:6:73:941:1973#0/1”, and the new format used by CASAVA version 1.8 like “@EAS139:136:FC706VJ:2:2104:15343:197393 1:Y:18:ATCACG”) [9].

**Figure 1:**
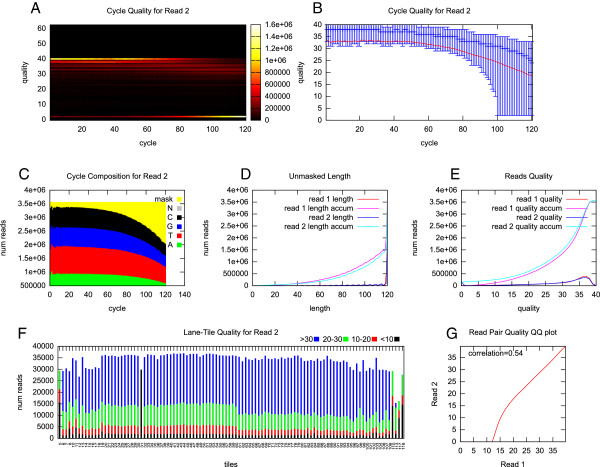
**Charts for read quality assessment. A**: heatmap for read quality along read. **B**: box chart for read quality along read. **C**: base composition along read. **D**: read length distribution. **E**: read quality distribution. **F**: read quality on different tiles. **G**: correlation between two read ends.

The quality control pipeline begins with a quality assessment on raw reads. Program *ht_stat* generates QC report from raw sequence reads in different aspects. On each position of the sequence reads, a heat map and a box chart are presented with quality distribution (Figure 1AB). The stacked bar chart that represents the base composition on each position is shown at the same time (Figure 1C). The cycle-specific errors and the rapid-falling quality on reads tail can be viewed on these three charts. To find tile-specific problems like high error rate or low data production, a stacked bar chart shows the number of reads in different quality ranges using different color, each tile in one bar (Figure 1F). The other charts that show the distribution of reads with varied length and reads with varied quality can provide an overview of sequencing quality (Figure 1DE). For paired-end reads quality assessment, the *ht_stat* program is used to create separate charts for each end, and to calculate the correlation between reads quality of two ends (Figure 1G). All these results generated by *ht_stat* program are written to a series of tab-delimited plain-text files, which can be visualized using *ht_stat*_draw.pl, or any spreadsheet software like Microsoft Excel or LibreOffice Calc.

After the assessment of sequence reads quality is obtained, low quality reads should be removed. The HTQC tool kit provides four different programs that include *ht_tile_filter*, *ht_trim*, *ht_qual_filter* and *ht_length_filter*, to perform reads filtration. The *ht_tile_filter* is designed to remove reads from problematic tiles that may not be reliable due to sequencing chip quality; the *ht_trim* cuts low quality bases at the beginning or the end of the reads until the quality score reaches a given threshold; the *ht_qual_filter* remove reads with low quality and the *ht_length_filter* remove short reads. When only one end of the paired-end reads is of acceptable quality, it is stored in a separate file. The cutoff value of these programs, such as the thresholds on the minimum reads quality or minimum read length are user defined.

## Results and discussion

### Workflow demonstration

To demonstrate the function of HTQC, a paired-end sequence data of human gut metagenome was used as an example. To reduce the time cost, one tenth from a total of 35,625,015 paired-end reads were randomly picked. The reads length was 120bp. The quality assessment was performed using *ht_stat*, which shows the reads quality in a series of charts that were described above in Implementation. When quality assessment was done by base position, there was a gradual decrease of reads quality towards the 3’-end (tail) that can be observed in Figure 1A and 1B. The tail trimming would be routinely applied to cut the low quality reads using the program *ht_trim*. In Figure 1C, there was at least 10% of reads that have an invalid nucleotide sequence represented by contiguous Ns. The bad reads that contained these Ns can be filtered with the program *ht_length_filter* or *ht_qual_filter*. When the quality assessment was done by tiles, we observed tiles 5, 31, 110, 113, 117, 118 produced reads with very low quality (Figure 1F) that can be removed using *ht_tile_filter*. For the quality assessment of any paired-end reads, if the quality of read 2 was worse than read 1, such quality imbalance can be picked up by *ht_stat* (Figure 1G).

### Program run-time efficiency

**Figure 2:**
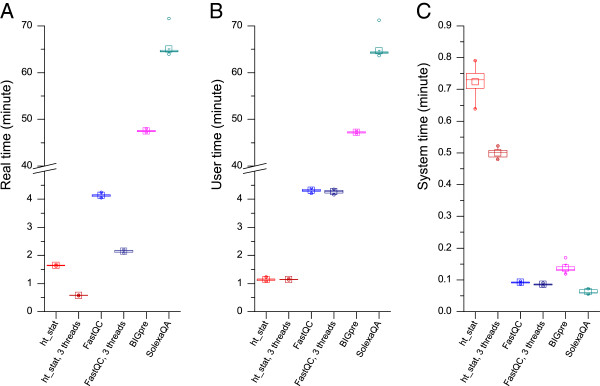
**Time cost of *ht_stat *and other QC software. A**: real time; **B**: user time; **C**: system time. The meaning of “real”, “user” and “system” time is described in the chapter of program run-time efficiency.

To compare the time cost of HTQC with existing tools, quality assessment on the above dataset was performed using *ht_stat*, SolexaQA version 1.12, FastQC version 0.10.1 and BIGpre version 2.0.2. The benchmark was performed using a Dell PowerEdge server with two AMD Opteron 2427 CPUs (12 cores in total). The server’s operation system was Fedora Linux 14. For SolexaQA, it by default only uses a part of the input sequences from each tile, thus we input the size of the test dataset (3,559,988 reads) as the tile sample size to ensure all reads were used. For BIGpre, due to its inability to parse the newest version of header format produced by CASAVA 1.8, or to work on quality records with “@” character, we modified its source code to allow it work properly with the test dataset. The SolexaQA and the modified BIGpre programs were executed using Perl version 5.12.4 contained within the Fedora Linux system. The C++ source code of *ht_stat* was compiled using GNU GCC version 4.5.1 with optimization flag “-O2”. For the performance test of multiple threads, benchmarks for *ht_stat* and FastQC were carried out in two groups: one benchmark using 1 worker thread, the other benchmark using 3 worker threads. Therefore, we have six benchmarks in total: one each for SolexaQA and BIGpre, two each for *ht_stat* and FastQC. All benchmarks were run 20 times repeatedly to overcome any random disturbance of the computer system, and the amount of real time (elapsed time in real world), system time (CPU cost in system mode) and user time (CPU cost in user mode) were recorded using the *time* command that is a part of the Linux base system. When compared with the other two Perl programs, both benchmarks of *ht_stat* used one order of magnitude fewer amount of user time, which showed the advantage of C++ language. The parallel comparison with FastQC, a Java program, *ht_stat* was about three times faster in both single thread and three threads test (Figure 2A). However, the higher amount of system time in our program shows the cost of the use of threads, which indicates our parallel processing model needs further improvements (Figure 2C). When comparing the two benchmarks of *ht_stat*, the time cost of the three-threaded benchmark is only one third of the one-threaded benchmark, which indicates the efficiency of multiple threads (Figure 2A). Furthermore, there is no significant difference in the amount of user time and system time between 1-thread and 3-thread benchmarks of *ht_stat* (Figure 2B), which indicates the use of more threads does not produce additional system cost.

## Conclusions

The HTQC tool kit provides convenient utilities for Illumina sequencing QC. It can process sequencing data faster than the existing tools, and generates quality assessment report both in plain-text and graphical representation, which can help in making decisions about further reads filtration. The HTQC package also provides four programs that can perform reads filtration using different methods. Unlike previous tools in which only single filtration method is allowed, user can choose the method they prefer to remove the low quality reads, and combine several filtration methods in any order.

### Availability and requirements

Project name: HTQC

Project home page: https://sourceforge.net/projects/htqc

Operation system: Linux, potentially any POSIX compliant system.

Other requirements: GNU Glib http://ftp.gnome.org/pub/GNOME/sources/glib, pkg-config http://www.freedesktop.org/wiki/Software/pkg-config, CMake http://www.cmake.org, Perl http://www.perl.org, Gnuplot http://gnuplot.info

Programming languages: C++, Perl

License: GNU GPL version 3 or later

## Abbreviation

QC: Quality control.

## Competing interests

The authors declare that they have no competing interests.

## Authors’ contributions

XY implemented the program; FL, XX, JZ and JW provided test data; BLZ, DL, FQZ and XY wrote the manuscript. All authors read and approved the final manuscript.
